# Intraoperative Anaphylaxis in Response to Hemostatic Agents With Protein Derivatives

**DOI:** 10.7759/cureus.9881

**Published:** 2020-08-19

**Authors:** S. Elliott Holbert, Darren Patel, Tony Rizk, Nahu G Dimitri, Micah Jones

**Affiliations:** 1 Surgery, Edward Via College of Osteopathic Medicine, Blacksburg, USA; 2 Orthopedics, Edward Via College of Osteopathic Medicine, Blacksburg, USA; 3 Interventional Radiology, Edward Via College of Osteopathic Medicine, Blacksburg, USA; 4 Emergency Medicine, University of Medicine and Health Sciences, Basseterre, KNA; 5 Orthopedic Surgery, LewisGale Medical Center, Salem, USA

**Keywords:** anaphylaxis, hemostatic powder spray, topical thrombin, alpha gal, gelatin, allergy, tick-borne disease

## Abstract

Topical hemostatic agents are commonly used in a wide variety of surgical procedures to assist in hemostasis. However, the use of these agents is not without risk as many contain biologically active agents derived from human and animal products that have the potential to cause adverse reactions. This case report covers a 44-year-old man with a history of alpha-gal syndrome who was scheduled for an open reduction and internal fixation of a left distal radius fracture. Alpha-gal syndrome is characterized by an IgE-mediated type 1 hypersensitivity reaction to a mammalian oligosaccharide epitope. Patients with this condition have a history of a past tick bite and subsequent development of an allergic reaction to mammalian protein products, most notably red meat. The patient had concerns about products used during surgery and potential reactions based on his allergy. The intent of this case report is to promote physician awareness of the widespread use of mammalian products in surgical hemostatic agents and potential immunogenic reactions. By increasing awareness of the alpha-gal syndrome, the goal is that medical device companies will actively disclose product components that could potentiate these adverse reactions and continue to develop alternative agents.

## Introduction

Hemostasis is an integral part of surgical procedures, and it is important to be cognizant of potential life-threatening complications. Many medical specialties utilize hemostatic interventions in a variety of situations, such as preventive measures, technical considerations, transfusions, or administrations of systemic or topical hemostatic agents [1]. Various patient comorbidities can complicate the use of certain hemostatic agents; thus, a thorough review of medical history is recommended to determine which hemostatic method would be best suited to each individual patient. Topical hemostatic agents are typically used when other traditional hemostatic techniques are insufficient at controlling bleeding, or at the end of procedures to promote coagulation prior to the closure [1]. Some of the more commonly used hemostatics include gelatin and thrombin-based products. In short, these products work by providing factors suitable to promote platelet adhesion and aggregation to stimulate the natural coagulation cascade [2]. One concern with the use of these products is a lack of physician awareness of their individual components, some of which include animal or human protein products and can lead to immune-related complications [1,2]. The following case report entails narrowly averted intraoperative anaphylaxis in response to surgical hemostatic agents in the setting of routine operative management of a left distal radius fracture.

## Case presentation

**Figure 1:**
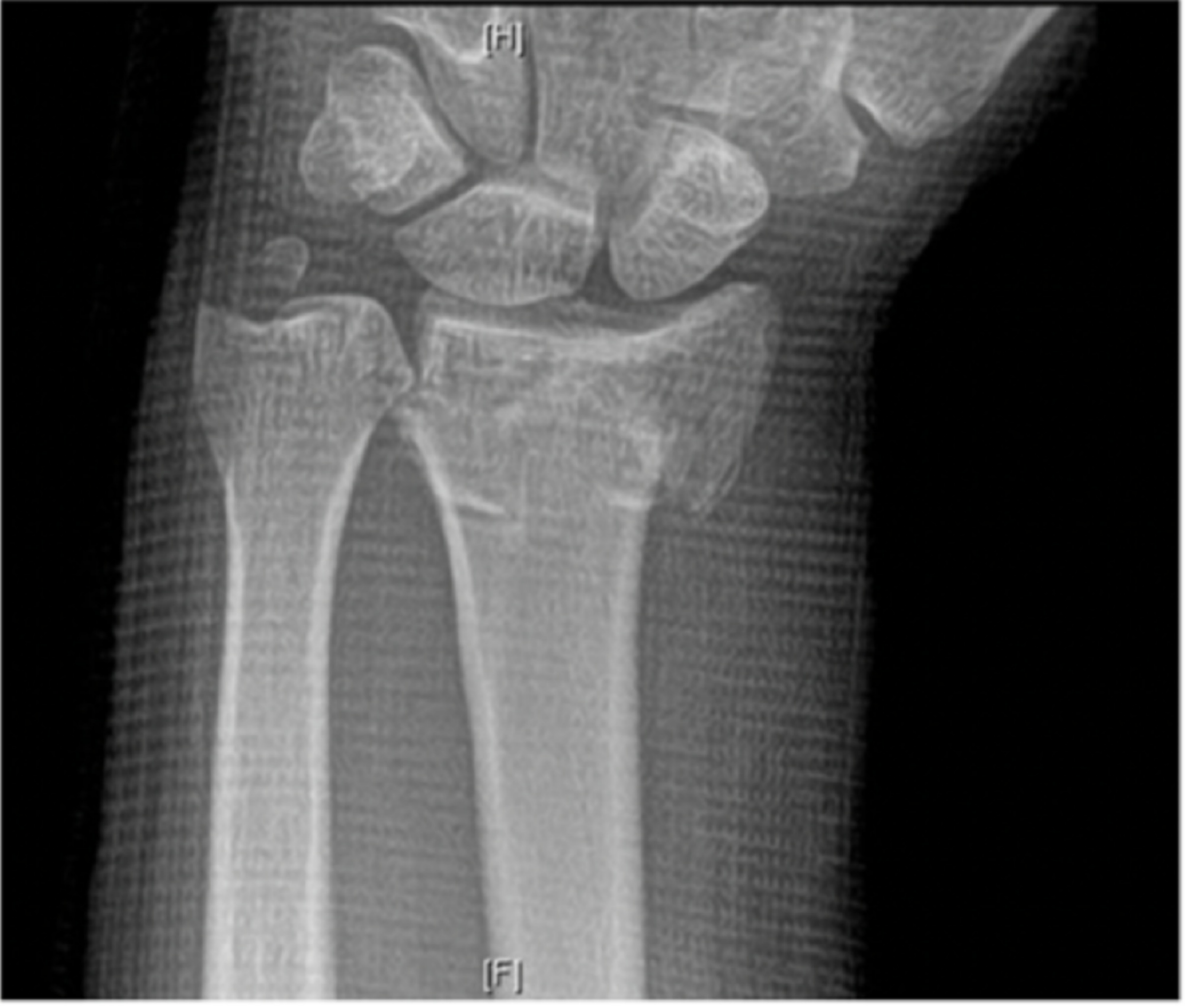
Anterior-posterior (AP) X-ray of the left wrist showing comminuted distal radius fracture with dorsal angulation of the distal fragment and a displaced ulnar styloid fracture

**Figure 2:**
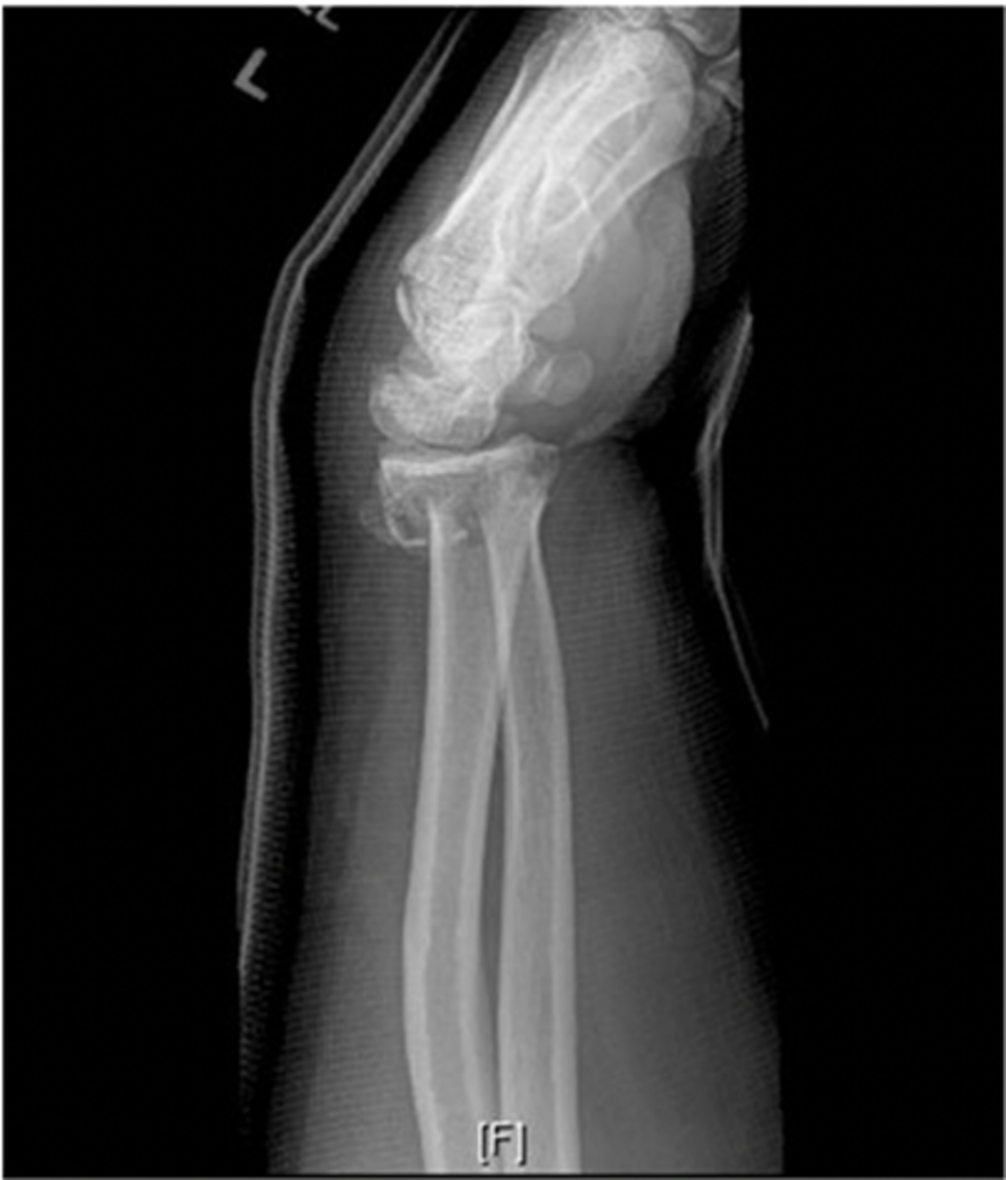
Lateral X-ray of the left wrist showing comminuted distal radius fracture with dorsal angulation of the distal fragment and a displaced ulnar styloid fracture

A 44-year-old Caucasian male presented to the orthopedic clinic with pain on the volar aspect of his forearm after a fall onto his hand at a construction site. The pain was constant, non-radiating, and mildly improved with non-steroidal anti-inflammatories. The patient’s neurovascular status was intact, and radiographs demonstrated a fracture of the left distal radius and displaced ulnar styloid fracture. Initially, closed reduction and casting were attempted. Follow-up radiographs demonstrated signs of loss of reduction, shortening of the radial height, dorsal angulation, and ulnar positive variance with a change from initial postreduction x-rays (Figures 1, 2). 

Possible causes for loss of reduction include a complex fracture pattern with dorsal comminution, loss of radial height, and initial displacement of the fracture prior to reduction. If left as casted, the fracture would heal as a nonunion requiring an opening wedge osteotomy with open reduction and internal fixation (ORIF) and bone grafting or possibly ulnar shortening [3]. In addition, the patient’s status as a current smoker raised concern for future nonunion. As such, surgical treatment was recommended and agreed upon by the patient. The patient’s goal was to regain as close to normal function as possible due to injury to his dominant hand, and occupation as a skilled laborer.

The patient underwent ORIF of the left distal radius fracture with tenotomy of the brachioradialis tendon and extensor tendon tenolysis of a three-week-old fracture. For such procedures, the senior author elects to place Gelfoam® (Pfizer, New York, NY) or thrombin spray prior to closure to assist with postoperative hematoma prevention and bleeding.

During the surgical procedure, Surgifoam® (Ethicon, Somerville, NJ) was placed in the wound after definitive surgery and immediately prior to wound closure over the distal radius and volar plate to help provide hemostasis and reduce potential postoperative blood loss. Approximately 5,000 units of Recothrom® (Baxter Healthcare, Deerfield, IL) topical recombinant thrombin spray was also prepared as an additional hemostatic agent, but not utilized during the case. 

Critically, the surgeon recalled acute facts about the chosen hemostatic agents and relevant patient history. The patient’s past medical history includes alpha-gal syndrome and allergy with anaphylactic reaction to mammalian meat-based products. Secondly, the surgeon had reviewed the product information sheets for both Recothrom and Surgifoam, noting use of mammalian byproducts in both hemostatic agents.

Recothrom is derived from a Chinese hamster ovary cell line, and the product information contains a warning that the product may contain hamster or snake proteins [4]. Similarly, Surgifoam has porcine-derived gelatin products used in its manufacturing [5]. Both hemostatic agents contain products that are derived from mammals and therefore may have served as potential triggers for intraoperative anaphylactic reaction due to the patient’s pre-existing alpha-gal syndrome.

Due to the aforementioned facts, irrigation for the Surgifoam was held and the Surgifoam was able to be removed as one piece from the patient’s wound. Afterwards, the wound was thoroughly irrigated with two liters of saline. No thrombin spray was utilized secondary to the surgeon’s discretion to avoid potential intraoperative anaphylactic reaction. The remainder of the procedure proceeded as scheduled. As a precaution, 25 mg of diphenhydramine and 4 mg of dexamethasone, in addition to the preoperative 8 mg dexamethasone dose, were administered to prevent potential anaphylactic reaction. The patient was informed of the potential adverse effects of Gelfoam with respect to his alpha-gal allergy in the postoperative anesthesia care unit. He was monitored closely, had an uneventful postoperative recovery, and was discharged later that afternoon in stable condition.

**Figure 3:**
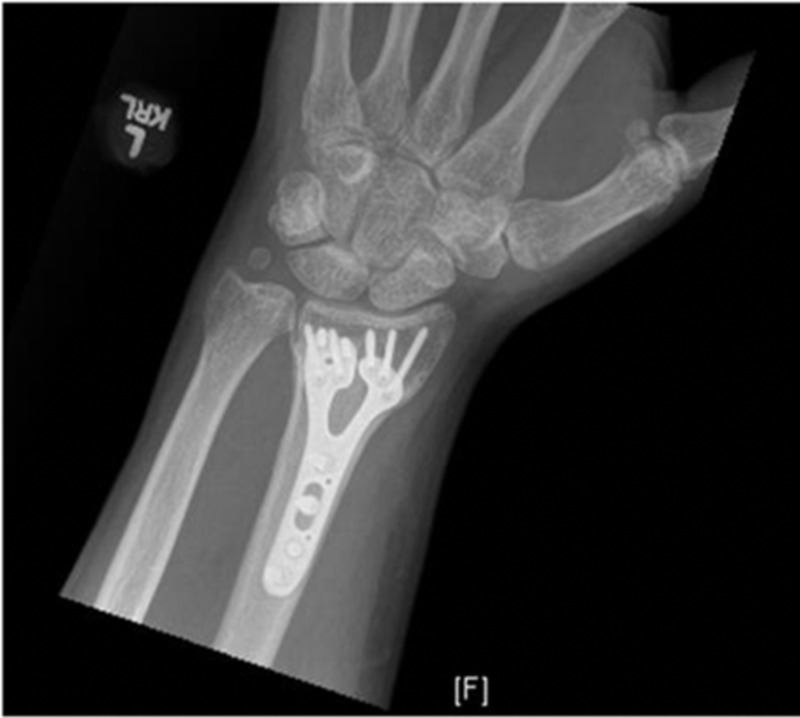
Anterior-posterior (AP) X-ray of the left wrist showing volar plate fixation with a healed distal radius fracture and small displaced ulnar styloid fracture which appears stable from previous X-rays

**Figure 4:**
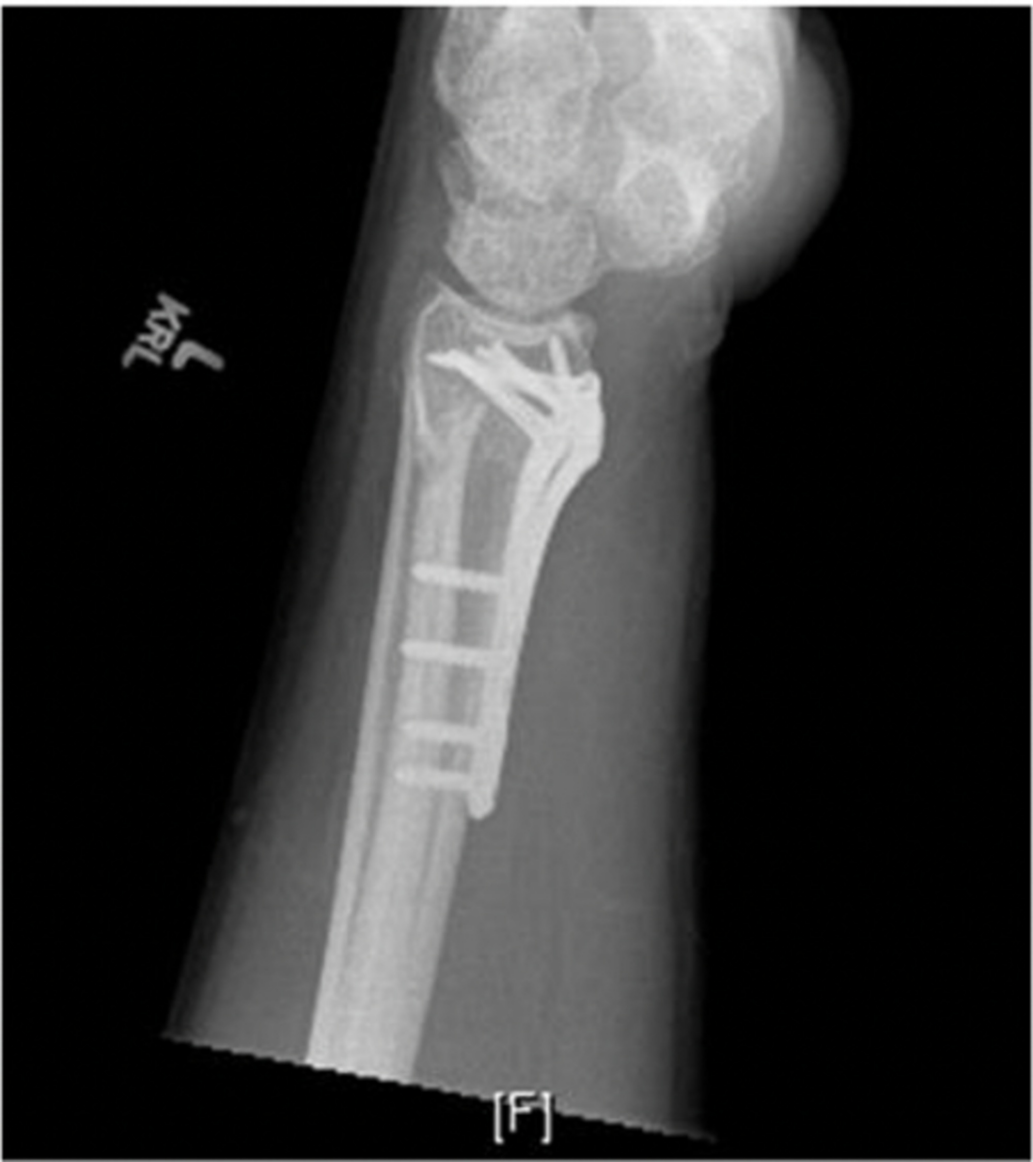
Lateral X-ray of the left wrist demonstrating volar plate fixation with a healed distal radius fracture and small displaced ulnar styloid fracture with appears stable from previous X-rays

The patient was seen at routine intervals for follow-up and noted no adverse reactions postoperatively. Postoperative radiographs demonstrated appropriate bone healing and uncomplicated volar plate fixation (Figures 3, 4). On the physical examination, no neurovascular deficiencies were noted, and the patient had near full range of motion of the wrist with 50 degrees of flexion and 40 degrees of extension with no pain at the fracture site.

## Discussion

Alpha-gal syndrome is an allergic tick-borne disease that stems from the production of IgE antibodies against a mammalian oligosaccharide epitope, Galα1-3Galβ1-(3)4GlcNAc-R (α-Gal). This carbohydrate, which is present on the tick salivary glycoproteins and non-catarrhine mammalian tissues, produces Th2 cell-mediated immunity and an IgE-mediated hypersensitivity anaphylactic reaction that typically manifests three to six hours after red meat consumption. Patients do not typically show any symptoms until at least two hours after consumption, which is atypical for allergic reactions. The initial manifestations include gastrointestinal symptoms and urticaria, progressing to angioedema or even anaphylaxis [6]. This allergic reaction is also observed in association with certain drugs, such as immediate anaphylaxis when administering intravenous cetuximab, as well as anaphylaxis in response to future tick bites. In the United States, *Amblyomma americanum* is associated with the allergy, while *Ixodes ricinus* and *Ixodes holocyclus *are implicated in Europe and Australia, respectively. 

Normally, the IgG and IgM antibodies to α-Gal that are produced by gut bacteria are protective against several pathogens, such as tuberculosis and malaria. However, the production of IgE antibodies towards α-Gal following a tick bite in some individuals results in a loss of oral tolerance to food allergens, with subsequent anaphylactic reactions in response to α-Gal [7]. When past studies attempted to determine why certain individuals developed this reaction to α-Gal, common risk factors were noted in the literature: family history of atopy, certain ABO blood group compositions, cat ownership, alcohol consumption, exercise, age, and certain medications. Although the factors mentioned are associated with the development of α-Gal, there are still many undiscovered mechanisms that work in combination with exposures to produce the disease [7].

Topical hemostatic agents are widely used in surgical settings to aid the body’s natural coagulation cascade by promoting platelet adhesion, aggregation, and fibrin clot formation. Hemostatic agents, such as Gelfoam, contain dermal gelatins produced from boiled porcine or bovine skin, horns, and hoofs [2]. Gelfoam is also commonly used as an embolic agent in interventional radiology to induce intrinsic thrombus formation and partial vessel occlusion with eventual blood flow restoration to healed tissues, such as in the case of pelvic trauma [8]. Traditionally, thrombin products were derived from the bovine or human plasma that carried the intrinsic risk of transmitting blood-borne pathogens or prion diseases [9]. The genome of a Chinese hamster ovary cell line has been utilized to consistently produce stable recombinant human prethrombin-1 protein, which is a precursor to α-thrombin, the active component in thrombin products.

Patients with α-Gal have been reported to experience intraoperative anaphylactic reactions to the gelatin components of certain topical hemostatic agents. This reaction is immediate rather than the delayed reaction observed with red meat consumption, presenting with rapid onset hypotension, tachycardia, elevated airway pressure, and an erythematous rash. A rapid removal of the agent with irrigation is imperative, followed by immediate infusions of epinephrine, corticosteroids, and antihistamines.

Lied et al. described a patient with a suspected anaphylactic reaction to Surgiflo® (Ethicon, Somerville, NJ) that occurred during the last phase of an atrial septal defect repair. In this patient, Surgiflo was placed over the sutures on the outside of the right atrium and hemodynamic instability suddenly occurred. Vital signs became stable roughly 60 minutes after bolus injections of epinephrine and norepinephrine were given along with subsequent epinephrine infusion, corticosteroids, and antihistamines. The patient later had an allergological evaluation where he reported a history of red meat allergy and tick bites, and examination revealed high IgE antibody levels specific for alpha-gal [2]. Mozzicato et al. described another group of cases where three patients received either bovine or porcine valve replacements and went on to develop a spectrum of allergic reactions intra- or postoperatively. These reactions included extensive rash, angioedema, respiratory distress, hypotension, and anaphylaxis. Each patient was stabilized with a combination of antihistamines, steroids, and vasopressors. Two of the three patients noted an extensive history of tick bites and delayed allergic reactions related to the ingestion of red meat. All three patients were found to have significantly elevated levels of IgE specific for alpha-gal [10]

Overall, the incidence of intraoperative anaphylactic reactions is rare, ranging from 1:4,000 to 1:25,000 [2]. Since the α-Gal syndrome is an IgE-mediated type-1 hypersensitivity reaction, in vitro immunoglobulin testing can be utilized for detection. A significant correlation has been shown between a positive skin prick test with gelatin and elevated levels of α-Gal IgE in the patient's serum who have these types of reactions [2]. Acceptable alternative topical hemostatic agents that can be employed in these patients include those that are constructed from oxidized regenerated cellulose, cyanoacrylate adhesives, and polyethylene glycol. These plant and polymer products are free of allergens making them safer to use in those with allergic reactions to α-Gal. 

## Conclusions

With the rising incidence of alpha-gal syndrome, this presentation highlights the importance of taking a thorough allergy history during preoperative patient interactions and emphasizes physician awareness of potential reactions including, but not limited to, intraoperative anaphylaxis. If the suspicion of the allergy is high, formal allergy testing should be performed to confirm the diagnosis and to tailor the appropriate hemostatic agent for the procedure. Physicians should be aware of the ingredients used in their choice of hemostatic agents, with consideration of potential allergens. Patients suffering from α-Gal syndrome would benefit from topical agents composed of plant and polymer products to avoid the risk of inducing anaphylaxis. In the event of anaphylaxis, rapid recognition, agent removal, and prophylaxis are crucial to facilitate appropriate management and ensure patient safety.
